# Towards a more robust non-invasive assessment of functional connectivity

**DOI:** 10.1162/imag_a_00119

**Published:** 2024-03-28

**Authors:** Britta U. Westner, Jan Kujala, Joachim Gross, Jan-Mathijs Schoffelen

**Affiliations:** Donders Institute for Brain, Cognition and Behaviour, Radboud University, Nijmegen, The Netherlands; Department of Cognitive Neuroscience, Donders Institute for Brain, Cognition and Behaviour, Radboud University Medical Center, Nijmegen, The Netherlands; Department of Psychology, University of Jyväskylä, Jyväskylä, Finland; Institute for Biomagnetism and Biosignal Analysis, University of Münster, Münster, Germany

**Keywords:** MEG, EEG, source reconstruction, beamformer, functional connectivity

## Abstract

Non-invasive evaluation of functional connectivity, based on source-reconstructedestimates of phase-difference-based metrics, is notoriously non-robust. This isdue to a combination of factors, ranging from a misspecification of seed regionsto suboptimal baseline assumptions, and residual signal leakage. In this work,we propose a new analysis scheme of source-level phase-difference-basedconnectivity, which is aimed at optimizing the detection of interacting brainregions. Our approach is based on the combined use of sensor subsampling anddual-source beamformer estimation of all-to-all connectivity on a prespecifieddipolar grid. First, a pairwise two-dipole model, to account for reciprocalleakage in the estimation of the localized signals, allows for a usableapproximation of the pairwise bias in connectivity due to residual leakage of“third party” noise. Secondly, using sensor array subsampling, therecreation of multiple connectivity maps using different subsets of sensorsallows for the identification of consistent spatially localized peaks in the6-dimensional connectivity maps, indicative of true brain region interactions.These steps are combined with the subtraction of null coherence estimates toobtain the final coherence maps. With extensive simulations, we compareddifferent analysis schemes for their detection rate of connected dipoles, as afunction of signal-to-noise ratio, phase difference, and connection strength. Wedemonstrate superiority of the proposed analysis scheme in comparison tosingle-dipole models, or an approach that discards the zero phase differencecomponent of the connectivity. We conclude that the proposed pipeline allows fora more robust identification of functional connectivity in experimental data,opening up new possibilities to study brain networks with mechanisticallyinspired connectivity measures in cognition and in the clinic.

## 1 Introduction

The brain is considered to operate as a network of interacting, functionallyspecialized regions. The development and application of analysis tools to probethose interactions in the healthy human brain from non-invasive electrophysiologicalmeasurements has been an active area of research in the past few decades. Part ofthat work is grounded in the notion that interregional interactions may be reflectedby statistical dependencies between band-limited signal components that can bepicked up from locally activated brain areas. One way to quantify this so-calledfunctional connectivity is to estimate some measure of relative phase consistency orphase synchrony (Varela et al., 2001), forinstance using the coherence coefficient, or a derived metric (Bastos & Schoffelen, 2016). From a mechanisticpoint of view, it has been hypothesized that consistent phase differences ofoscillatory processes facilitate neuronal interactions by virtue of a mutualtemporal alignment of cycles of increased neuronal excitability (Bonnefond et al., 2017;Fries, 2005,^2015^). In sum,connectivity estimates based on phase synchrony are a valuable metric in cognitiveneuroscience.

It is commonly agreed that, for interpretability, connectivity estimates should beassessed at the source level. This is because connectivity estimates are invariablyconfounded by spatial leakage (Schoffelen &Gross, 2009). Promising work from the early 2000s developed (Gross et al., 2001) and successfully applied(e.g.,Pollok et al., 2005;Schoffelen et al., 2005) the Dynamic Imaging of CoherentSources (DICS) technique, a frequency domain version of a beamformer for sourcereconstruction, to identify networks of phase-synchronized brain regions based onthe strong physiological periodicities during smooth finger movements in healthyparticipants. Further studies focused on synchrony at the frequency of Parkinsonianor essential tremor in clinical populations (Polloket al., 2004;Timmermann et al.,2003). In the decades following this early work, the research communityhas also started studying envelope correlations of band-limited signals instead ofphase synchrony. This latter metric has been successfully used to identifyproperties of networks predominantly during the brain’s resting state,yielding a body of literature with well interpretable and consistent findings (Baker et al., 2014;Brookes et al., 2011;Colclough et al., 2016;de Pasquale etal., 2016;Hipp et al., 2012).Despite ongoing methodological work to improve source reconstruction (Dalal et al., 2006;Hillebrand et al., 2012;Kuznetsova et al., 2021;Nunes et al.,2020;Woolrich et al., 2011) andnovel phase synchrony based connectivity metrics (Aviyente et al., 2011;Ghanbari &Moradi, 2020;Vinck et al., 2011),neuroscientific findings employing phase synchrony seem to be more scarce and lessconsistent (Colclough et al., 2016;O’Neill et al., 2018).

Assuming that metrics based on phase differences tap into fundamental mechanisms ofbrain organization and communication (Bonnefond etal., 2017;Fries, 2005,^2015^), then why is it seemingly so difficult tofind converging evidence across studies? One reason for this might be that themethodological adversities are larger than commonly assumed (Bastos & Schoffelen, 2016;He et al., 2019;Palva etal., 2018). One of those difficulties is spatial leakage, both fromsecond-party and third-party sources, that is, seed-based leakage involving one ofthe two interacting sources or leakage into both interacting sources from a thirdsource, respectively. Not being able to estimate and remove this leakage renders thelower bound of the true connectivity unknown. Proposed techniques for leakagecorrection, on the other hand, can be too aggressive and also compromise or evenremove the signal of interest. Furthermore, data quality might further impede thereliable estimation of phase difference: low signal-to-noise ratio (SNR) mighthinder the reliable identification of seed regions of interest, while SNRdifferences across conditions occlude the interpretation of connectivity, since theestimation of phase-based connectivity measures is sensitive to SNR changes.

In this paper, we propose a new method that tackles these problems. We propose toaddress the issue of suboptimal region of interest (ROI) or seed selection throughconsideration of the full 6-dimensional all-to-all connectivity source space, usinga two-dipole constraint beamformer. We further propose an estimation of the nullcoherence which approximates the bias in the coherence estimate and can be used tocorrect the output. Finally, we reduce estimation bias by aggregating over theresults of many source reconstructions using sensor array subsampling, therebycreating a more stable and robust estimate.

In the following, we will first introduce beamforming for source reconstruction andexplain the problem of spatial leakage in more detail. Then, we will outline thecomponents of our proposed beamforming approach.

### 1.1 Beamformers for source reconstruction

Non-invasive electrophysiological measurements (electric potential differencesfor electroencephalography (EEG) or magnetic fields (gradients) formagnetoencephalography (MEG)) reflect a mixture of the temporal activationprofiles from neural and non-neural sources. To estimate the neural sources thatcontribute to the spatiotemporal mixture in the observed signals, sourcereconstruction techniques can be applied. These techniques have developed into avaluable tool for the analysis of non-invasive electrophysiological signalsobtained during cognitive experiments. Solving the so-called inverse problem bycombining a forward model with additional assumptions, source reconstructiontechniques aim to build models of the spatiotemporal characteristics of theneural generators that underlie the measured signals, unmixing the observedchannel-level data. The biologically plausible forward model (or gain matrix)describes the spatial distribution of the observed signals, typically for a setof equivalent current dipole sources. The additional model assumptions arenecessary to constrain the number of solutions to the inverse problem, which inprinciple are unlimited. Adaptive beamformers are a class of sourcereconstruction techniques that do not a priori make explicit assumptions withrespect to the number or location of active sources, but rather assume theunderlying sources to be temporally uncorrelated. Usually, for each of a set ofpredefined source locations, a spatial filter is constructed under twoconstraints: 1) a unit gain constraint, which means that it should pass on allof the activity that originates from that specific location, and 2) a minimumvariance constraint, which minimizes the variance of the reconstructed activityat each location. Mathematically, this linearly constrained minimum variance(LCMV;Van Veen et al., 1997) spatialfilter is computed as follows:

$${\text{w}}^{⊤}(r)={\left[{\text{h}}^{⊤}(r){\text{C}}^{-1}\;\text{h}(r)\right]}^{-1}{\text{h}}^{⊤}(r){\text{C}}^{-1},$$ (1)

wherew(r)is the spatial filter atsource locationrand⊤refers to the transpose operation.h(r)is the sourcelocation-specific gain vector (which can be thought of as a spatialfingerprint), and${\text{C}}^{-1}$is the mathematical inverse of the channel covariance matrix. As an alternativeto the channel-level covariance matrix, one can use a complex-valuedcross-spectral density (CSD) matrix, based on the channel Fourier coefficientsfor a given frequency bin, resulting in the DICS algorithm (Gross et al., 2001).

Beamformers have gained prominence as one of the most popular sourcereconstruction techniques because they typically provide relatively robustreconstructions of neural activity without the need of sophisticated parametertweaking (Westner et al., 2022). However,some limiting factors exist with respect to functional connectivity. In thefollowing, we will present the typical distortions when source reconstructingfunctional connectivity, as well as our approach to mitigate these.

### 1.2 The effect of signal leakage on source connectivity estimates

In the context of connectivity estimation, an important concept is that of signalleakage. This refers to the fact that each location’s estimated activityreflects an unknown mixture of the true activity at this location and signalcontributions from distant noise sources of both neural and non-neural origin.Mathematically, this can be shown as detailed below.

Considering the generative model of the sensor-level data, the sensor signalsreflect a summation of the underlying source signals, each multiplied by theirspatial fingerprint:

$$X=\sum_{i=1}^{I}h(r_{i},q_{i})s_{i}+N.$$ (2)

Here,Xis a number-of-channels bynumber-of-observations matrix with complex-valued Fourier coefficients,his the real-valued gainvector for a dipolar source at location$r_{i}$and with orientation$q_{i}$,and${\text{s}}_{i}$is a 1 by number-of-observations source activity vector, here assumed to becomplex-valued, that is, to reflect both amplitude and phase for theobservations.Nis a number-of-channelsby number-of-observations matrix, reflecting the non-brain noise in the measureddata.

Assume that we have computed a pair of spatial filters,${\text{w}}_{1}$and${\text{w}}_{2}$,and we use these spatial filters to compute an estimate of the source-levelFourier coefficients:${\hat{s}}_{1}={\text{w}}_{1}^{⊤}\text{X}$, and${\hat{s}}_{2}={\text{w}}_{2}^{⊤}\text{X}$. From these estimates, one can compute a connectivity metric,for instance the coherence coefficient, for this dipole pair:

$$coh=\frac{\left|{\hat{s}}_{1}{\hat{s}}_{2}^{\text{H}}\right|}{\sqrt{\left({\hat{s}}_{1}{\hat{s}}_{1}^{\text{H}}\right)\left({\hat{s}}_{2}{\hat{s}}_{2}^{\text{H}}\right)}},$$ (3)

whereHdenotes the conjugate transposition. Note that for simplicity of notation, weomit the scaling with the number of observations, which drops out of theequation anyhow. We also note that a non-zero numerator in the equation abovesuggests linear dependence between the estimated sources 1 and 2. Below, weinspect this quantity, that is, the cross-spectral density estimate between thetwo sources, in more detail.

For the given pair of dipoles, and considering the data model$\text{X}={\text{h}}_{1}{\text{s}}_{1}+{\text{h}}_{2}{\text{s}}_{2}+\text{N}$withNnow reflecting all signalcontributions to the observed data that are not originating from the two dipolepairs-of-interest, we can express the cross-spectral density estimate betweenthe two dipoles as:

$$\begin{array}{ccc} {\hat{s}}_{1}{\hat{s}}_{2}^{\text{H}} & =\left(w_{1}^{⊤}X\right){\left(w_{2}^{⊤}X\right)}^{H}\\ & =\left(w_{1}^{⊤}\left(h_{1}s_{1}+h_{2}s_{2}+N\right)\right){\left(w_{2}^{⊤}\left(h_{1}s_{1}+h_{2}s_{2}+N\right)\right)}^{H}. & \end{array}$$ (4)

Introducing$g_{ij}$as a scalar value that results from computing the innerproduct between spatial filter$w_{i}$and gain vector$h_{j}$and which reflects the filter’s gain at locationifor a source originating from locationj,we obtain:

$${\hat{s}}_{1}{\hat{s}}_{2}^{\text{H}}=(g_{11}s_{1}+g_{12}s_{2}+w_{1}^{⊤}N){\left(g_{21}s_{1}+g_{22}s_{2}+w_{2}^{⊤}N\right)}^{H}.$$ (5)

When using an inverse algorithm with a typical unit-gain constraint,$w_{i}^{⊤}h_{i}=g_{ii}=1$, the above further reduces to:

$$\begin{array}{ccc} {\hat{s}}_{1}{\hat{s}}_{2}^{\text{H}} & =\left(s_{1}+g_{12}s_{2}+w_{1}^{⊤}N\right){\left(g_{21}s_{1}+s_{2}+w_{2}^{⊤}N\right)}^{H} & \\ & \begin{array}{l} =\left(s_{1}+g_{12}s_{2}\right){\left(g_{21}s_{1}+s_{2}\right)}^{H}+\left(s_{1}+g_{12}s_{2}\right)N^{H}w_{2}\\ \;\;\;\;+w_{1}^{⊤}N{\left(g_{21}s_{1}+s_{2}\right)}^{H}+w_{1}^{⊤}NN^{H}w_{2}. \end{array} & \end{array}$$ (6)

In other words, for a given dipole pair, the estimated cross-spectral density between two sources does not only depend on the sources’ true cross-spectral density, but is also affected by:

1. signal leakage from the other dipole-of-interest, specificallywhen$g_{12}$and$g_{21}$are non-negligible,*cf.*theleftmost term in the above equation
2. the interaction between the noise, projected through thespatial filter, and the sources’ activity,*cf.*the middle two terms in the aboveequation
3. the interaction between the projected noise at the location ofthe dipoles,*cf.*the rightmost term in the aboveequation

Note that the above reasoning is independent of the exact inverse algorithm used.The different types of leakage will also affect the estimates of the individualdipoles’ power. Leakage will always cause misestimation of metrics thatare derived from the estimated source level quantities. This also applies tospatial maps of connectivity, which are typically constructed using a limitednumber of predefined seed dipole locations. Local maxima in these spatial maps(which are either expressed as a difference between two experimental conditionsor in relation to a baseline) are then interpreted as regions that arefunctionally connected to the seed dipole. Irrespective of the specificconnectivity metric used, spatial structure in these maps due to leakage maylead to inference of false positive connections. Furthermore, true connectionsmay be missed altogether, if the seed dipoles have been misspecified by theresearcher.

### 1.3 Alleviating the effect of leakage

In order to address some of the problems associated with leakage, it has beenproposed to use connectivity metrics that disregard the interaction along thereal-valued axis (e.g., the imaginary part of coherency (Nolte et al., 2004) or the multivariate interactionmeasure (MIM,Ewald et al., 2012)), or toremove the instantaneous leakage originating from one or more dipoles prior toestimating the connectivity on the residuals (Brookes et al., 2012;Colclough etal., 2015;Hipp et al., 2012;Wens et al., 2015). Although theseadjustments avoid an overinterpretation of leakage-affected findings, thesensitivity to true signal interactions at small phase differences isdiminished. In more recent years, a few source reconstruction techniques thatmitigate this effect have been proposed (Hindriks, 2020;Ossadtchi et al.,2018). However, these leakage correction schemes do not eliminate thenecessity to compare the estimated connectivity against a well-defined baseline.This step is usually not straightforward since an appropriate baseline is notavailable: either because of differences in the signal specific to condition orsubject group (see, e.g.,Bastos &Schoffelen, 2016), or because of the absence of a baseline conditionaltogether (e.g., in single-group resting-state studies). Finally, in a contextwhere seed-based connectivity maps are evaluated, there is no guarantee that theseed regions have been appropriately specified.

In this work, we propose an analysis scheme of source-level connectivity (hereexpressed as the coherence coefficient), accounting as much as possible for theeffects of leakage but without a reduction in sensitivity for true interactionsat small phase differences. Moreover, we will derive estimates of a usable lowerbound of the estimated coherence between dipole pairs, which can be used as acorrection to more accurately evaluate spatial maps of connectivity, thusavoiding the issues associated with inappropriate or absent baseline conditions.Using extensive simulations, we show superiority of our analysis scheme incomparison to other approaches.

### 1.4 Proposed analysis approach

The analysis approach we outline in this paper consists of several elements: Wemake use of a two-dipole constraint beamformer (Brookes et al., 2007;Dalal et al.,2006;Moiseev et al., 2011;Schoffelen et al., 2008), approximateand correct for the estimated bias due to noise leakage, and embed the approachin a sensor array subsampling scheme. Below, we will discuss all those elementsin more detail.

#### 1.4.1 Two-dipole constraint beamformer and null coherence estimate

The approach is based on an all-to-all approach, where coherence is estimatedbetween all pairs of beamformer reconstructed dipoles defined on an evenlyspaced 3-dimensional grid, covering the entire brain. Using a two-dipoleconstraint in a beamformer formulation, we compute pairwise spatial filtersthat are not corrupted by zero lag correlations for the dipole pair underconsideration.^1^Abeamformer with two dipoles in its spatial passband has an identity gainconstraint:

$$W^{⊤}H=\left[\begin{array}{c} w_{1}^{⊤}\\ w_{2}^{⊤} \end{array}\right]\left[h_{1}h_{2}\right]=\left[\begin{array}{cc} g_{11} & g_{12}\\ g_{21} & g_{22} \end{array}\right]=\left[\begin{array}{cc} 1 & 0\\ 0 & 1 \end{array}\right].$$ (7)

As a consequence, the equation that expresses the estimated pairwise dipolarcross-spectral density reduces to the below equation, in analogy of themodel formulation as used in the previous section:

$${\hat{s}}_{1}{\hat{s}}_{2}^{\text{H}}=s_{1}s_{2}^{H}+s_{1}N^{H}w_{2}+w_{1}^{⊤}Ns_{2}^{H}+w_{1}^{⊤}NN^{H}w_{2}.$$ (8)

**Fig. 1.**
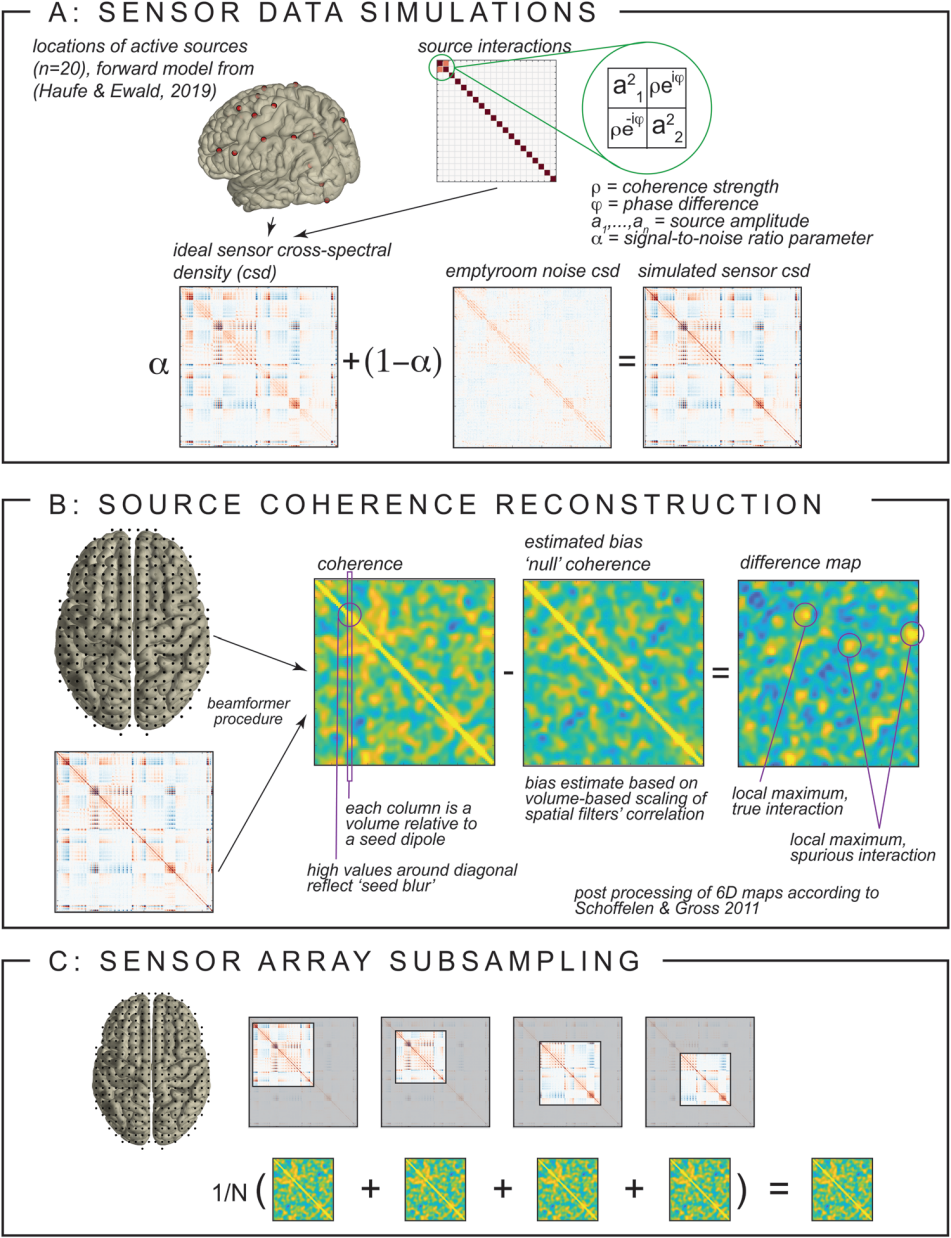
Key components of the algorithm and simulation. (A) Setup of sensordata simulation, illustrating the interacting and non-interactingsources and signal-to-noise ratio. (B) Estimation of the nullcoherence across space and the computation of the difference maps.(C) Illustration of the sensor array subsampling procedure with avarying number of sensors among realizations. Note that eachsubsampling iteration contains the coherence reconstructionprocedure described in (B), as well as (for the simulations only)the procedure described in (A).

Under the assumptions that the cross-terms between the noise (i.e., the partof the measured signal that does not originate from the locations ofinterest) and the considered sources are negligible, and if the noisecovariance is assumed spatially white (a scaled identity matrix), then theabove equation reduces to:

$${\hat{s}}_{1}{\hat{s}}_{2}^{\text{H}}=s_{1}s_{2}^{H}+\sigma w_{1}^{⊤}w_{2},$$ (9)

whereσis a scalar parameter. Under the described (yet most likely oftenviolated^2^)assumptions, if the true interaction strength between the two dipoles iszero, the expected value of$s_{1}s_{2}^{H}$ineq. 9will be zero (i.e., with alarge enough sample). Thus, the estimated cross-spectral density between thetwo sources may be approximated with a scaled version of the spatialfilters’ inner product,$\sigma w_{1}^{⊤}w_{2}$,where the scaling parameter is a function of the pair of source locations.From this follows, that the scaled spatial filter inner product can be usedas an approximation of the bias in estimated connectivity under theassumption of no interaction between the considered sources, an estimate wecall “null coherence.” Pragmatically, we propose to assume thescaling parameter to be fixed for a given seed dipole (i.e., keeping one ofthe dipoles in the pair fixed, and scanning through the dipole grid for theother dipole of the pair), and thus allow for its estimation by fitting aregression line through a two-dimensional point cloud, which reflects on onedimension the absolute value of the estimated cross-spectral density betweenthe seed dipole and all other dipoles, and on the other dimension theabsolute value of the inner product between the seed dipole’s spatialfilter and the other dipoles’ spatial filters. Repeating this fittingprocedure for all dipoles and normalizing by the product of the estimatedpower yields a 6-dimensional volume of null-coherence estimates, which canbe used to subtract from the estimated coherence (*cf.*Fig. 1B). The resulting 6-dimensionaldifferential map can subsequently be post-processed (e.g., thresholded) andinspected for local maxima, which might be indicative of truly interactingdipoles.

#### 1.4.2 Array subsampling

As will become clear below, these difference maps may still exhibit spatialnoise, resulting in false positive connections (i.e., local maxima that donot reflect interacting dipoles), and true connections being missed (i.e.,reconstructed connectivity between locations close to interacting dipolesnot presenting as local maxima). To further reduce the spatial noise in theimages, we propose a sensor array subsampling approach (Schoffelen et al., 2012;Westner, 2017;Westner et al., 2015). We estimate the 6-dimensionaldifferential connectivity map multiple times, each time using a differentrandom subset between 50 and 150 sensors for the reconstruction(*cf.*Fig. 1C). The rationale behind thisrepeated subsampling and subsequent aggregating of results is that thevariable noise across iterations will average out, while truly connecteddipoles will be present in most iterations. This might first seemcounterintuitive, since reconstructions with fewer sensors may have acompromised spatial resolution; however, the spatial noise will be variableacross reconstructions and thus average out, while the true interactionswill show up more consistently. This scheme is akin to the idea of EnsembleMethods in machine learning, such as Bagging (Breiman, 1996) or Random Forests (Breiman, 2001), where the iteractive subsampling of observationsand/or features helps reduce variance in the ultimate model. The aggregationof many weak learners leads to a strong model with reduced variance, or, inthe words ofBreiman (1996):“Bagging goes a ways toward making a silk purse out of a sow’sear, especially if the sow’s ear is twitchy.”

## 2 Methods

All simulations and reconstructions were performed in MATLAB (version 2021b) on aLinux operated High Performance Compute cluster, using FieldTrip (Oostenveld et al., 2011) and custom written code.

### 2.1 MEG sensor data simulations

MEG sensor space complex-valued data matrices were simulated from source spaceactivity, based on a 275-channel axial gradiometer CTF system, as a combinationof an ‘ideal’ sensor-level signal data matrix$X_{s}$and a noise data matrix$X_{n}$.These$N_{\text{sensor}}\times N_{\text{observation}}$matrices reflect the Fourier coefficients(i.e.*,*amplitude and phase information) computed for agiven frequency. For the noise matrix we used a multitaper spectral estimate ofa frequency band centered around 10 Hz from a 50 second empty room measurement,recorded at the Donders Centre for Cognitive Neuroimaging. The empty room datawere segmented into 1 second epochs and spectrally transformed, using amultitaper smoothing parameter of±4Hz (7 tapers per segment), which resulted in a268×350noise matrix. The number 268 reflects the number of activeSQUIDs at the time of the empty room measurement, 350 the number of observations($N_{\text{epochs}}\times$$N_{\text{tapers}}$). The signal data matrix was constructed using the generativemodel$X_{s}=\mathrm{HS}$, using a precomputed forward modelH(see below), and an$N_{\text{source}}\times N_{\text{observation}}$matrixS. The source signals weresimulated using MATLAB’s mvnrnd function, generating multivariateGaussian data, with a mean of 0, and a parametrized covariance (cross-spectraldensity) matrix, defined as:

$$\begin{array}{l} \text{diag}\left(a,a,\left(1-a\right),\ldots ,\left(1-a\right)\right)\left[\begin{array}{ccccc} 1 & \rho {\text{e}}^{\text{i}\varphi } & 0 & \cdots & 0\\ \rho {\text{e}}^{-\text{i}\varphi } & 1 & 0 & \cdots & 0\\ 0 & 0 & 1 & \cdots & 0\\ \vdots & \vdots & \vdots & \ddots & \vdots \\ 0 & 0 & 0 & \cdots & 1 \end{array}\right]\\ \;\;\;\;\;\;\;\;\;\;\;\;\text{diag}\left(a,a,\left(1-a\right),\ldots ,\left(1-a\right)\right), \end{array}$$ (10)

whereρreflects the intended coherence coefficient between the first two sources, andφreflects the phase difference.areflects a relative amplitude parameter, determining the relative amplitude ofthe connected dipoles in relation to the other active sources such that therelative strength between connected dipoles and active sources can be computedasa/(1−a),that is, a relative amplitude of 0.8 yields the connected dipoles being fourtimes stronger than the other active sources. The procedure for simulating thesensor space data is illustrated inFigure1A. For gain matrixH, we used aprecomputed forward model, as described inHaufeand Ewald (2019)and the Biomag conference 2016 data analysischallenge (seehttps://bbci.de/supplementary/EEGconnectivity/BBCB.html). Briefly,source locations were sampled from a cortical segmentation-based triangulatedmesh, originally consisting of 2004 positions. A three-shell boundary elementmethod (BEM) had been used to compute the forward solution for the 2004 dipoleswith an orientation perpendicular to the cortical sheet, using Brainstorm (Tadel et al., 2011). For the simulationspresented here, sets of 20 positions were randomly selected from a subset of 820positions. This subset was created based on the norm of the gain vectors for theorientation-constrained dipoles placed at those positions: We excluded candidatelocations for which the sensor array was relatively insensitive, for example,deep dipoles in the midline, or dipoles with an unfavorable orientation. Thematrices$X_{n}$and$X_{s}$were scaled with the Frobenius norm of their respective cross-spectral densities ($XX^{H}$)and linearly combined using:

$$X=\sigma X_{s}+(1-\sigma )X_{n},$$ (11)

whereσis a parameter that determines the signal-to-noise ratio.Table 1summarizes the relevant parameters for thesimulations and the values used to explore the different reconstructionapproaches.

**Table 1.** Simulation parameters.

Parameter	Values
# of active sources	20 in 100 different configurations
# of observations	350
σ , signal-to-sensor-noise	0.5*, 0.6
a , amplitude relation	0.5*, 0.7, 0.8*
ρ , coherence coefficient	0, 0.2, 0.3, 0.4, 0.5, 0.6, 0.7, 0.8
π , phase difference	0, (2/17)π , (4/17)π , (8/17)π , (16/17)π

Values marked with asterisks denote values for which the outcomes arereported in theSupplementary Material.

### 2.2 Beamformer source reconstruction and coherence estimation

For source reconstruction we used a forward model defined on a regularly spaced3-dimensional dipole grid (with a spacing of 8 mm). The brain compartment ofthis grid consisted of 4416 dipoles and was defined by the same anatomical MRIas the one used for the simulations’ forward model. For thereconstructions’ forward model, we used a realistic single shell model asimplemented in FieldTrip (Nolte, 2003).Our detailed analysis required the computation of$4416^{2}$pairs of spatial filters for many iterations of sensor array subsamples (we used100 subsamples per simulation) over 8000 parameter combinations. Thus, we had toestimate over 15 trillion spatial filters in total. We wrote custom code for theefficient computation of the spatial filters and the derived coherence. Allbeamformers were computed with FieldTrip’s fixedori constraint, whichcomputes a fixed orientation forward model for each dipole, based on themaximization of the beamformer’s output power (Sekihara & Nagarajan, 2008). The mathematicalinverse of the cross-spectral density matrices was estimated from the sensorsignals without applying regularization.

### 2.3 Evaluation criteria for the full simulation

We compared our subsampling approach to three other well-adopted beamformingapproaches: 1) a traditional single dipole beamformer, 2) a two-dipolebeamformer, and 3) a beamformer with a geometric correction scheme (Wens et al., 2015). We assessed theperformance of the different approaches on the simulated scenarios with respectto the correct identification of the true interacting dipole pair (the hitrate). We evaluated the hit rate with respect to coherence strength and phasedifference, as well as against the number of false positives using theFree-response Receiver-Operating-Characteristic (FROC). To further motivate ourapproach and show some results in more detail, we also include an‘illustrative example’.

### 2.4 Real data analysis

To test our method on real data, we analyzed data from a single subject,performing isometric extension of the left wrist. An earlier analysis of thesame data (Schoffelen et al., 2008)focussed on the identification of brain areas that are synchronized to theelectromyogram (EMG) in the beta frequency range. The data were acquired asdescribed inSchoffelen et al. (2008),and all subjects included in the study gave written informed consent accordingto the Declaration of Helsinki. Due to methodological limitations, highlightedboth in the current paper and the referenced work, a cortically seeded coherenceanalysis did not produce convincingly interpretable results. For that reason,the previous work identified the implicated brain areas beyond contralateralprimary motor cortex (cM1), using cortico-muscular coherence (CMC) analysis incombination with a two-dipole beamformer constraint, suppressing the correlatedsignal leakage from cM1. With the CMC approach, it was shown that ipsilateralcerebellum and sensorimotor areas were synchronized with the EMG in the betafrequency range. Here, we applied the subsampling technique to evaluatecortically-seeded coherence, in relation to the null-coherence estimate. First,we used dynamic imaging of coherence sources (DICS,Gross et al., 2001) to compute CMC between theelectromyogram and the source space MEG data at this subject’s optimalcoherence frequency (24 Hz). We then used the resulting location of maximumcoherence (contralateral primary motor cortex) as a seed for the subsequentanalysis. Next to analyzing the cortico-cortical coherence using the subsamplingapproach, we computed seed-based imaginary coherence using a beamformer with ageometric correction scheme, the difference between coherence and null coherenceas described in the Illustrative Example. For the subsampling, we used 250randomizations, with a random number of sensors between 40 and 120 (note thatthe data were obtained using a 151-channel MEG system). Source reconstructionwas performed on a 4 mm grid (resulting in 25815 sources), using asubject-specific multisphere model as volume conduction model. For visualizationpurposes, we computed the relative difference between the average (acrosssubsamples) of the estimated coherence and the estimated null coherence.

## 3 Results

### 3.1 Illustrative example and null coherence estimation

**Fig. 2.**
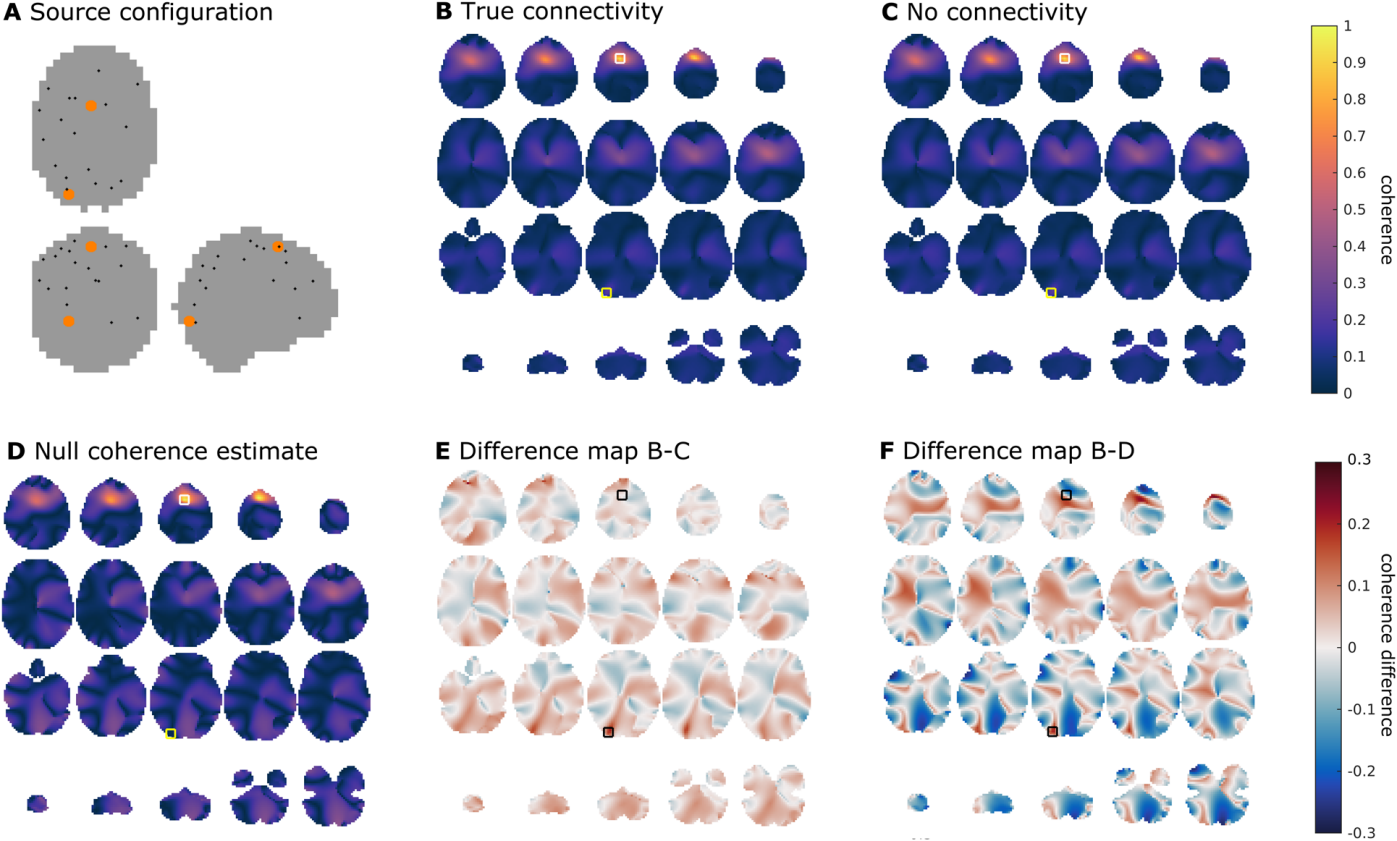
Illustrative example. (A) Spatial configuration for simulation. Shown are20 randomly selected dipole locations of active sources (small blackdots) and the two truly interacting sources (bigger orange dots). (B)Estimated coherence for true connectivity using a single dipolebeamformer. The white square denotes the seed, coinciding with one ofthe interacting sources. The yellow square denotes the location of theconnected dipole. (C) Same as (B), but with no underlying interaction.(D) Estimate of null coherence for the same data. (E) Difference map of(B) and (C), black squares denote the interacting dipoles. (F)Difference map of (B) and (D).

This section illustrates our proposed approach.Figure 2Ashows the spatial configuration of one instantiation ofthe simulation, where 20 dipole locations were randomly selected to reflect theactive sources. Two of these sources (the bigger, orange dots in the figure,here denoted as a medial superior frontal (MSF) and left occipital (LO) source)reflect the interacting dipoles. To illustrate the potential issues related tospatial leakage, we start by investigating different seed-based maps. In thisexample, we simulated the interaction to be at a phase difference of(8/17)πand the coherence strength to be 0.5. For illustrationpurposes, we computed these seed-based results on a 4 mm grid, but for thereconstruction of all pairwise interactions we used an 8 mm grid. For thisexample, we also simulated data using identical source parameters as for theabove simulations, apart from the coherence strength, which was set to zero.This simulation was intended to reflect a perfect baseline, where everythingexcept the interaction strength was kept constant. We start the illustrationusing a traditional single dipole beamformer.Figure 2Bshows the seed-based estimate of coherence for trulyinteracting sources, using as a seed the grid position that was closest to theMSF source (indicated with a white square).Figure2Cshows an estimate of the coherence for the scenario in which thedipoles were not connected. Both estimates are dominated by the well-known seedblur, but also show a small local maximum in the vicinity of the LO source(indicated with a red square) for the case of the true connectivity (Fig. 2B). The difference image (Fig. 2E) shows an effective suppression ofthe leakage close to the seed location. Yet, there is considerable spatialstructure in the residual image, and although there is a local maximum in thevicinity of the LO source, there are also other maxima in this image that may bemistaken for interacting sources. In many practical situations, an appropriatebaseline condition is not available. This motivated us to estimate the“null” coherence based on the scaled inner product of the spatialfilters (as described above), assuming this scaling to be fixed for a given seeddipole, and the noise to be spatially white and uncorrelated with the sources.Figure 2Dshows the computed nullcoherence for our illustrative example. The null coherence map shows structurewith a higher amplitude than the baseline condition inFigure 2C, and thus, the difference map between thecoherence and null coherence (Fig. 2F) alsoexhibits more structure (i.e., with a substantially higher overall amplitude)than the difference map with the baseline condition (Fig. 2E). Specifically, the seed blur inFigure 2Fdoes not seem to be very wellaccounted for given the difference map’s local maximum in the vicinity ofthe MSF seed region.

Before exploring the usage of different beamformer analysis schemes to improvethe connectivity results fromFigure 2, letus note thatFigure 2considered asituation in which the seed dipole for the connectivity estimation was wellchosen, that is, it coincided roughly with one of the truly interacting sources.In the analysis of experimental data, seed locations are not known a priori,thus one might happen to choose locations that are not truly interacting. Inthis case, the high spatial structure in the null coherence maps is replicatedeven for non-interacting seeds, which evidently would be problematic for realdata analysis. This effect is illustrated inSupplementary FigureS1.

### 3.2 Two-dipole beamformer and array subsampling

**Fig. 3.**
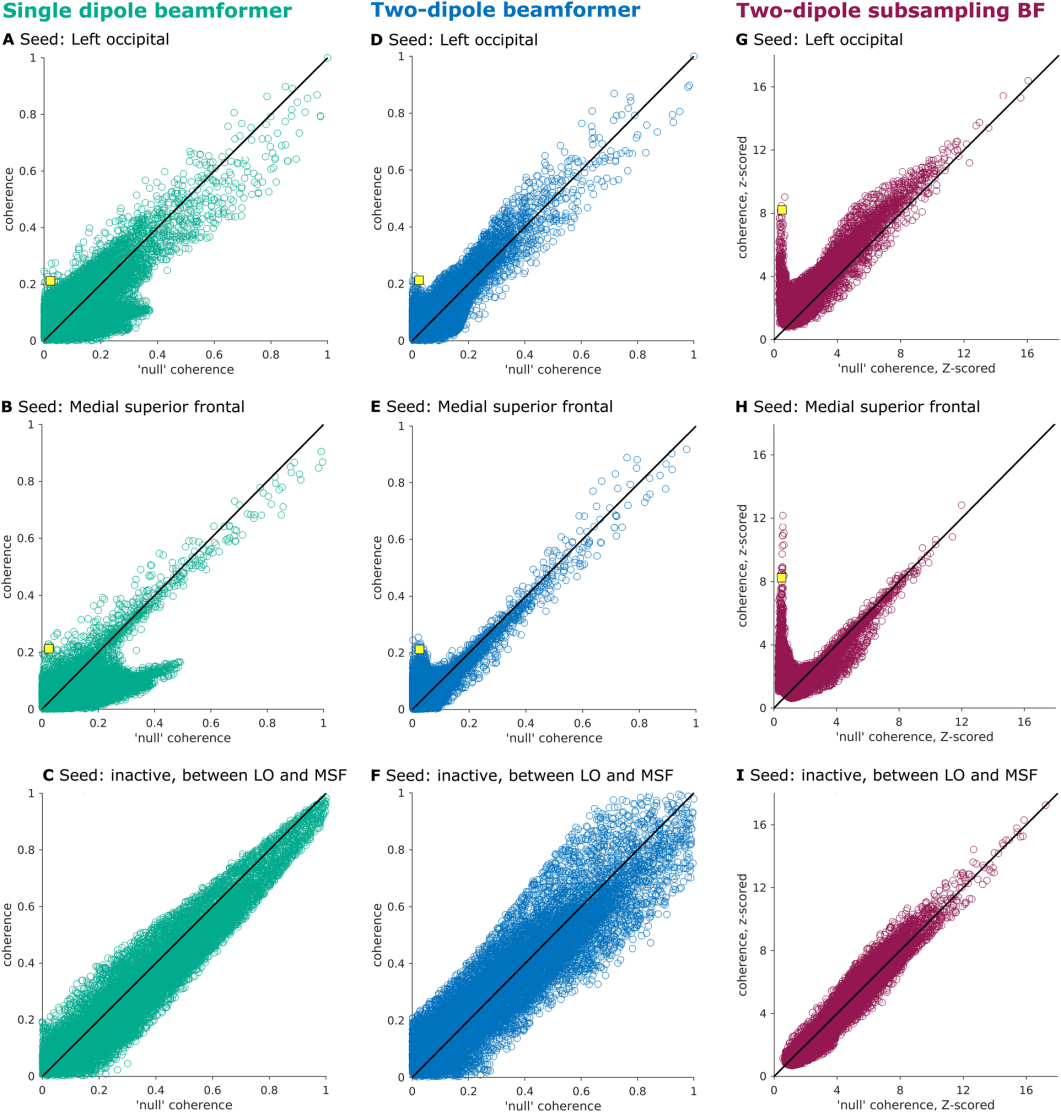
Comparing different beamformer approaches. (A-C) Single dipole beamformerwith (A) seed close to truly interacting source in left occipital cortex(LO), (B) seed close to truly interacting source in medial superiorfrontal cortex (MSF), and (C) seed in a non-active dipole located on theline between interacting dipoles LO and MSF. (D-F) Two-dipolebeamformer. (G-I) Two-dipole beamformer with array subsampling. Thevalues for the interacting dipole pair are highlighted with the yellowsquare.

At this point, one may argue that the suggested null coherence estimate isimpractical to use, given the large amount of residual noise in the differenceimages (*cf.*Fig. 1andSupplementary Fig. S1).In other words, the spurious connectivity estimated between two locations ispoorly approximated just by computing the spatial leakage of projected spatiallywhite sensor noise, at least when using a single dipole beamformer formulation.As motivated in theintroductionsection,the use of a two dipole-constraint in the beamformer formulation may reduce someof the leakage terms inequation 6,leading to a null coherence estimate that is better behaved. In addition, sensorarray subsampling allows for multiple (although possibly degraded) estimates ofthe true structure in the data, while unstructured noise is averaged out whenaggregating those estimates. Let us further investigate if the scaled spatialfilter inner product might be an appropriate estimate for spurious sourceinteractions when using those alternative beamformer approaches.Figure 3Arevisits the results fromFigure 2F, plotting the estimated nullcoherence (x-axis) against the estimated coherence (y-axis) for all dipoles inthe stimulation with the values for the interacting dipole pair highlighted withthe yellow square.Figure 3B and Cshow theresults for the same single dipole beamformer approach but with the other trulyinteracting source and a source between the two truly interacting sources asseeds, respectively. Ideally, for non-interacting dipoles, the data pointsshould cluster on a line around the diagonal, while the data point(s)corresponding to the truly interacting dipoles should be clearly above thediagonal. Comparing the single dipole beamformer (Fig. 3A and B) with the two-dipole beamformer (Fig. 3D and E) for the truly interacting dipoles suggeststhat, overall, the data points cluster more nicely around the diagonal line inthe two-dipole beamformer case.Figure 3G-Idepict the results of the subsampling approach. To this end, the average acrosssubsamples of the estimated coherence and null coherence was normalized with thestandard deviation of their difference. Here, the subsampling boosts thedetectability of the interacting dipole pair, by making it stand out clearlyfrom all other dipole pairs. Also, for seed dipoles in inactive andnon-interacting locations (bottom row), the spread of the data points around thediagonal is much more comparable across the different seed dipoles for thesubsampling-based reconstruction. In contrast, when no subsampling is used, thedeviations from the diagonal are substantially larger for the inactive seeddipole as compared to the active and interacting seed dipoles. This suggeststhat the magnitude of the spatial noise in the difference images variesconsiderably, depending on the choice of the seed dipole, and that the approachof array subsampling mitigates this effect by aggregating the results of manydifferent noise realizations.

### 3.3 Evaluating all-to-all pairwise coherence

To formally evaluate how the spurious spatial structure in the seed-basedconnectivity maps interacts with accurate detectability of the trueinteractions, we constructed and evaluated the all-to-all pairwise coherencematrix (Schoffelen & Gross, 2011).Here, each of the dipoles in the grid serves as a seed dipole to all otherdipoles. After the subtraction of an estimate of the null coherence, theresulting 6-dimensional volume of difference in coherence is thresholded, usinga relative threshold keeping theN%largest values. We explored the following values of N, with the correspondingnumber of unique supra threshold edges in parentheses: 5% ($9.8\times 10^{5}$),1% ($1.95\times 10^{5}$),0.5% ($9.8\times 10^{4}$),0.1% ($1.95\times 10^{4}$),0.05% ($9.8\times 10^{3}$),0.01% ($1.95\times 10^{3}$),0.005% (975), 0.001% (195), 0.0005% (98).

The thresholded maps are subsequently analyzed for the presence of clusters ofspatially connected dipoles in 6-dimensional space. Such clusters are consideredto reflect a potential long-distance interaction if they consist of two dipoleassemblies that are spatially distinct from each other. Clusters that containauto-connections, that is, dipoles that are present in both assemblies of theconnection, are discarded from further inspection. If the simulated interactingdipoles fall within the identified clusters, it is considered a hit. Allremaining clusters are considered false positives. It should be noted that thenumber of false positives evidently will increase with a decreasing thresholdwhen using a relative thresholding scheme as we do here. The total number offalse positives further depends on the blurriness of the spatial noise and thedegree of auto-connectedness in the data.

**Fig. 4.**
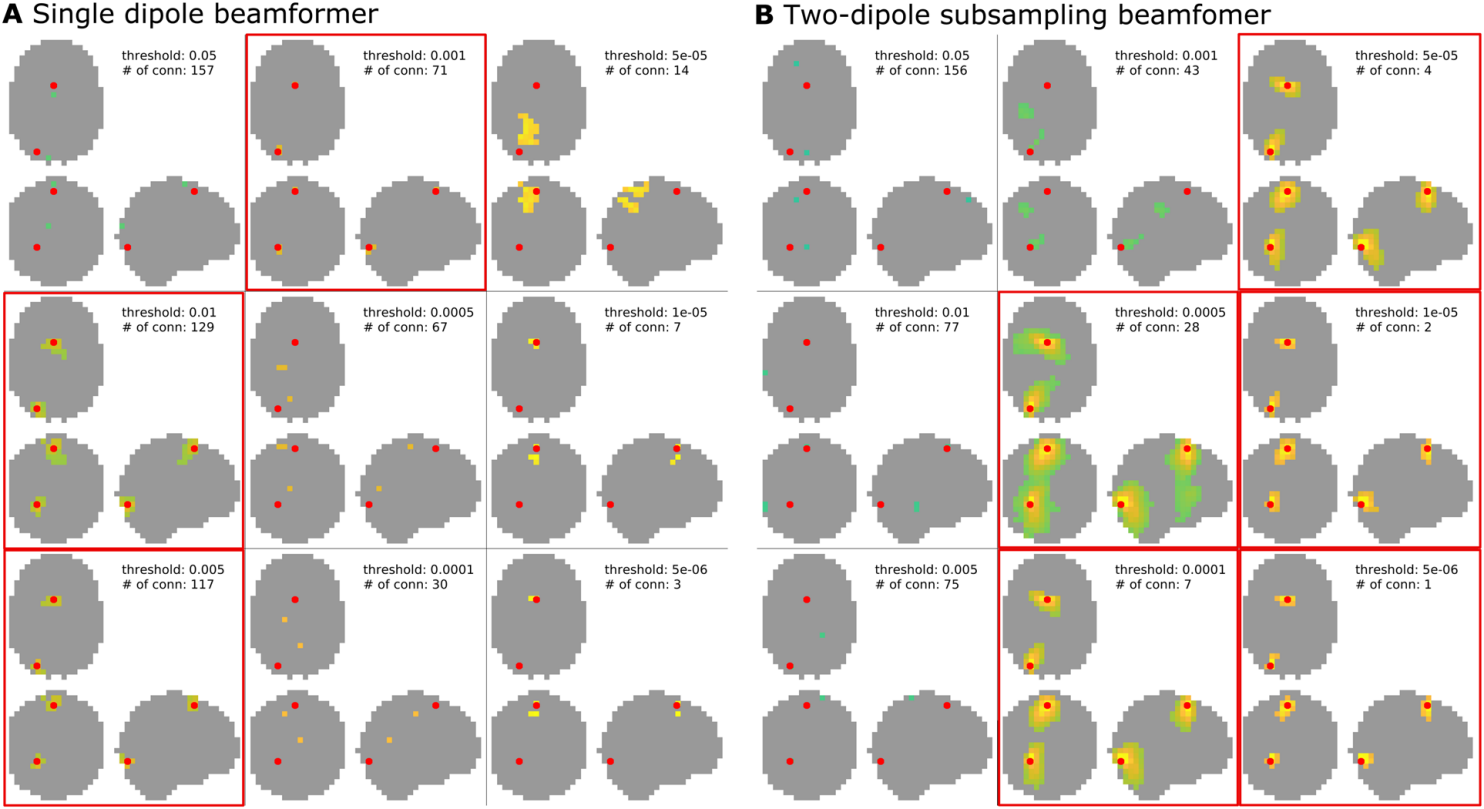
All-to-all pairwise coherence. Shown are the results at different clusterthresholds for the single dipole beamformer (A) and the two-dipolebeamformer with array subsampling (B). Each result also lists the numberof identified connections. Thresholds at which the truly interactingdipole pair was successfully identified are marked by a red frame.

Figure 4shows the clusters with thesmallest distance to the simulated interacting dipole pair, and the number ofdistinct connections, for each of the different thresholds applied. Using asingle dipole beamformer (Fig. 4A), thetrue connection can be correctly identified in three out of the ninethresholding schemes (marked with a red frame). This, however, comes at theexpense of additional false positive connections, ranging in number from 70 to128. Thus, in this relatively favorable context—where coherence is largeand the phase of the interaction is close to 90 degrees, that is, with only aminor instantaneous correlation between the two sources without the potentialcorresponding distortion of the beamformer due to correlated sources—theactual connection may be correctly identified, but one has to be prepared toaccept an additional large number of false positives.

**Fig. 5.**
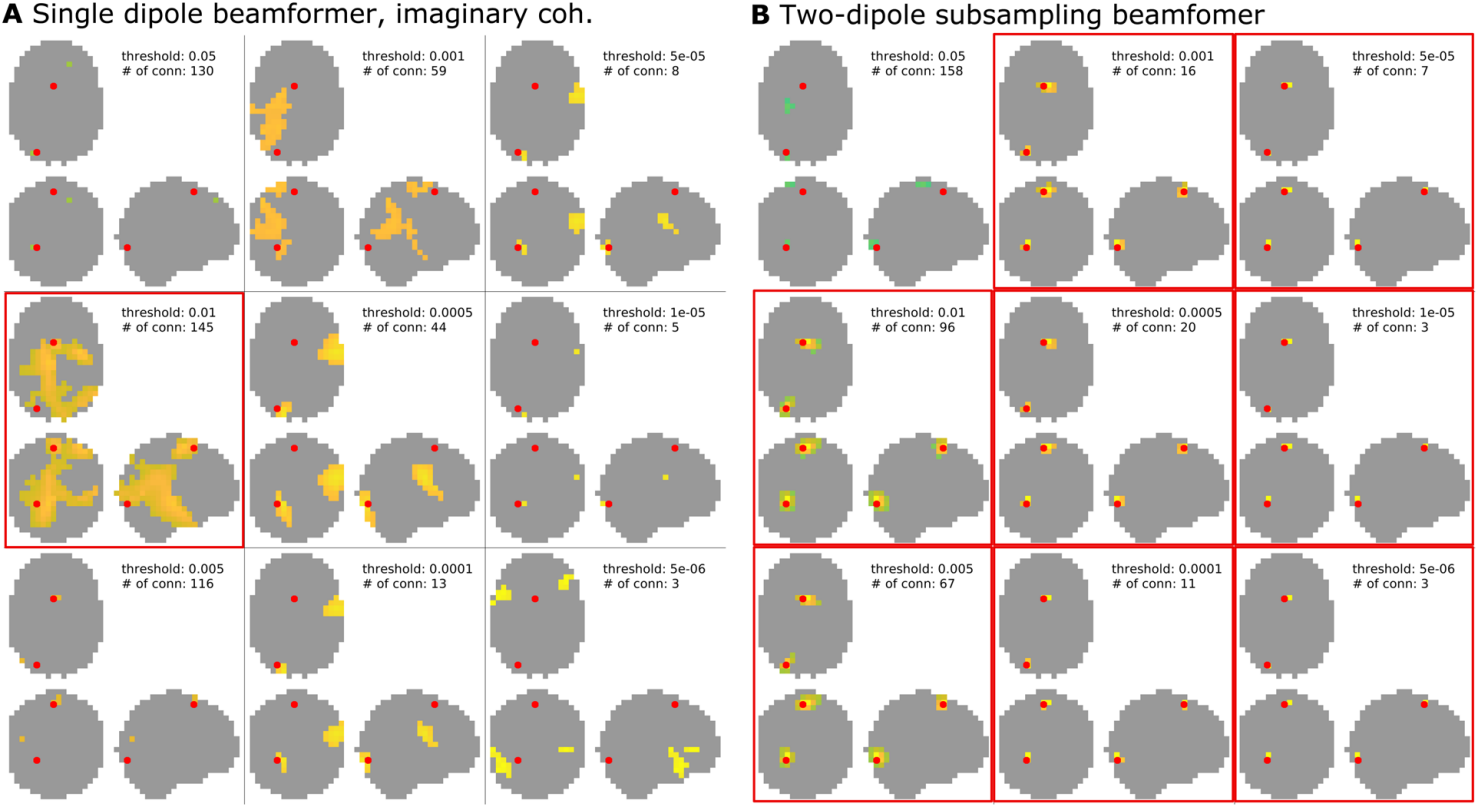
Impact of 0 degree phase shift. Results for interacting sources where thephase of the interaction is 0 degrees for the beamformer with thegeometric correction scheme, which focuses on the imaginary part ofcoherency (A) and the two-dipole beamformer with array subsampling (B).Each result also lists the number of identified connections. Thresholdsat which the truly interacting dipole pair was successfully identifiedare marked by a red frame.

Figure 4Bshows the spatial clustersclosest to the interacting dipole pair for the subsampling-based reconstruction.Here, the interacting sources are correctly identified in the five highestthresholding schemes (marked with red frames), with a considerable reduction inthe number of false positives as compared to the single dipole beamformer outputinFigure 4A. The number of false positivesdrops to one or none for the highest two thresholds applied. As an alternativeto analyzing the difference in coherence with an approximation of the estimatedbias under the assumption of no coherence, one can also investigate themagnitude of the imaginary part of the reconstructed coherency.Supplementary Figure S2in the Supplementary Material replicates the results fromFigure 2A(using a single dipole beamformer) for theimaginary part of coherency. With increasing threshold, the true connection canstill be reliably identified and the number of false positives drops to only twofor the highest two thresholds tested. Importantly, however, the usefulness ofthe imaginary part of coherency is limited to situations in which the phasedifference of the interaction is pointing away from 0 or 180 degrees.Figure 5shows the results for the sameinteracting dipole pair as in all previous examples, which are now interactingat a phase difference of zero. The subsampling approach (Fig. 5B) is still capable of detecting the interactingdipole pair at high thresholds, whereas the imaginary part of coherency approach(Fig. 5A) now fails at higherthresholds. Therefore, the subsampling approach with a two-dipole beamformerseems to work well regardless of the phase difference of the interacting dipolepair.

### 3.4 Full simulation results

**Fig. 6.**
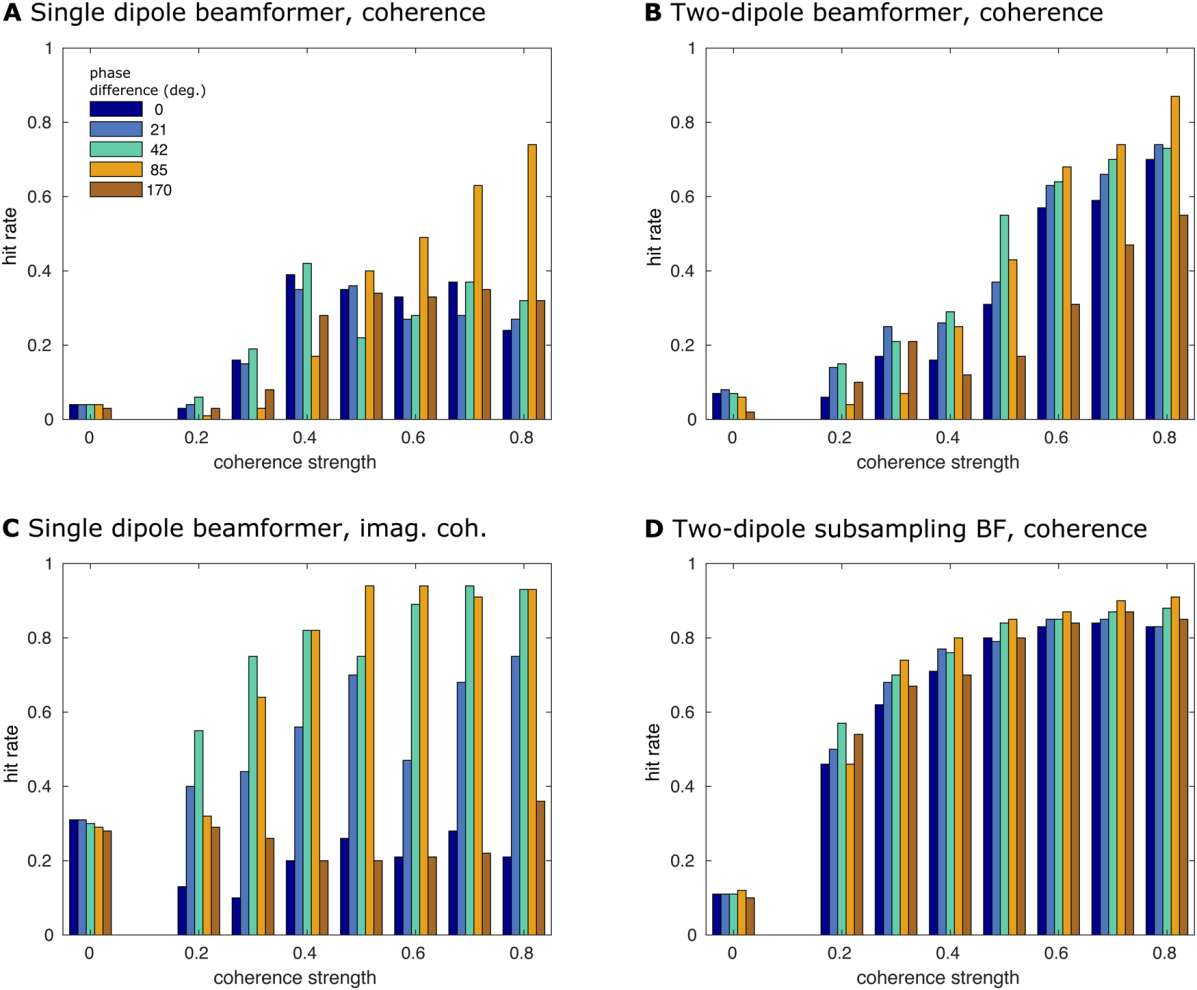
Detection rate for interacting dipole pairs. Results from the fullsimulation, showing the hit rates for the interacting dipole pair as afunction of simulated coherence strength and phase difference. Therelative amplitude of the interacting sources and the other sources wasa = 0.7, that is, the interacting sources were 2.333 timesstronger than the other active sources. The SNR was 0.6. (A) Traditionalsingle dipole beamformer. (B) Two-dipole beamformer. (C) Single dipolebeamformer using imaginary coherence. (D) Two-dipole beamformer witharray subsampling.

To test our proposed approach more thoroughly and to substantiate theillustrative results discussed so far, we employed an exhaustive simulation.Here, we compare the array subsampling two-dipole beamformer approach to threeother approaches: the traditional single dipole beamformer, the two-dipolebeamformer without subsampling, and a beamformer without subsampling, using ageometric correction scheme, proposed byWens etal. (2015). This correction scheme uses a spatial projectionheuristic to remove instantaneous leakage from a seed location’sestimated activity from all target locations’ estimated activity. Inpractice, this results in the real-valued component of the interaction betweenthe seed and target dipoles to be suppressed, leading to a purelyimaginary-valued coherency value. Therefore, in the below, we refer to this laststrategy as the reconstruction of the imaginary part of coherency. We evaluatethe source reconstruction results based on hit rate, that is, how often thechosen approach correctly identified the true interacting dipole pair.Figure 6shows the simulation results for arelative amplitude ofa=0.7, thus, the interacting sources were 2.333 times stronger thanthe other active sources (for the results fora=0.5anda=0.8, we refer the reader toSupplementary Figures S3 andS4, respectively). The results reported in the paper are based on asignal-to-sensor-noise ratio of 0.6; the results for an SNR of 0.5 are reportedinSupplementary FiguresS6-S8.

Figure 6depicts the hit rate as a functionof simulated coherence strength, and phase difference, for the differentreconstruction strategies. We first considered the situation in whichapplication of at least one of the thresholds<0.01%resulted in the detection of the dipole pair that was chosenfor the interaction (to define a hit, we allowed the summed distance of thesimulated dipoles to the closest voxel in the suprathreshold clusters to be atmost 2 cm). Overall, the performance of the single dipole approach (Fig. 6A) was quite poor, with the hitrate—as a function of coherence strength and phasedifference—rarely exceeding 40%. Only at unrealistically high coherencestrengths>0.6was the detection rate larger than 50%, and even then only atphase differences close to 90 degrees. The two-dipole approach (Fig. 6B) fared better, specifically forcoherence values larger than 0.4. The single dipole beamformer using imaginarycoherency (Fig. 6C) generally showed betterperformance, already at lower coherence values, but this performance was highlydependent on the phase difference of the interaction, where interactions with aphase difference close to 90 degrees were more readily detectable, reaching ahit rate of>90%in some situations. When the phase difference of theinteraction was close to 0 (or 180) degrees, however, the detection rate athigher coherence values was only slightly higher than for low coherencestrengths, compared with the single and two-dipole approach. The arraysubsampling beamformer (Fig. 6D) overallperformed best. Even though the maximum detection rate was not as high as insome situations using the imaginary part of coherency (i.e., coherence>0.5and phase difference close to 90 degrees), the detection rateat a moderate coherence of 0.3 already exceeded 60%, independent of the phasedifference. Thus, the array subsampling two-dipole beamformer outperforms theother approaches for almost all parameters, specifically considering the factthat physiologically realistic neuronal interactions are not constrained tophase differences close to 90 degrees, nor are those interactions restricted tohigh coherence values. The findings depicted inFigure 6are at large supported by the results of other amplitudeand SNR values, asSupplementary Figures S4andS6-S8illustrate.

**Fig. 7.**
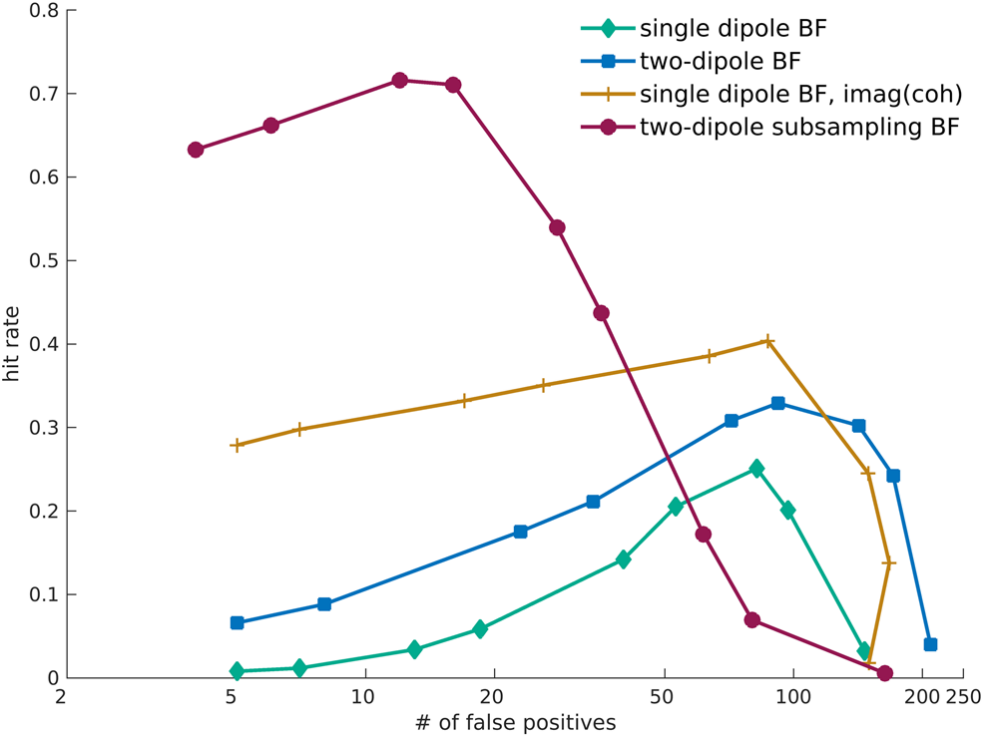
Free-response receiver-operating-characteristic. Hit rate plotted againstthe number of false positive connections at a relative amplitude ofa=0.7.

Notably, in the absence of simulated true coherence, the different reconstructionapproaches resulted in a variable amount of false positive connections in thedirect vicinity of a pair of activated dipoles (see the leftmost set of bars ineach of the panels inFig. 6). For theimaginary part of coherency, this type of false positive connection was presentin about 30% of the simulations, and for the proposed subsampling approach thepercentage of occurrence was about 10%. In general, the occurrence of falsepositives is the consequence of the fact that we used a relative thresholdingscheme to investigate the spatial structure of the reconstructed connectivitymaps. By construction, and irrespective of the numeric value of the connectivityestimates, the relative thresholding scheme always results in a collection ofsuprathreshold edges in the connectivity maps, which may be spatially clustered,and interpreted as interacting sources. Based on the spatial smoothness of theconnectivity maps, and the number of suprathreshold edges, the number of falsepositive connections will vary as a function of the chosen threshold.Figure 7shows the number of false positivesversus the hit rate in a so-called Free-responseReceiver-Operating-Characteristic (FROC), as a function of the detectionthreshold and for a relative amplitude ofa=0.7. On each of the lines, the threshold is increasing from leftto right. For all but the subsampling reconstruction method, theoptimal—yet still quite low—sensitivity was reached at a thresholdthat yielded close to 100 false positive connections on average. For thesubsampling reconstruction method, the highest sensitivity was compromised byabout 10 to 20 false positive connections on average. Although this still mayseem a rather high false positive rate, it is substantially lower than the falsepositive rate for the other approaches tested. The FROC curves for relativeamplitudes ofa=0.5anda=0.8can be found in the Supplementary Material (Fig. S5) and show verysimilar patterns. For an SNR of 0.5, the results are reported inSupplementary Figure S9and at large support the findings for an SNR of 0.6, except for at a lowrelative amplitude ofa=0.5, the only parameter combination for which the two-dipolesubsampling beamformer does not clearly outperform the other algorithms.

### 3.5 Real data results

**Fig. 8.**
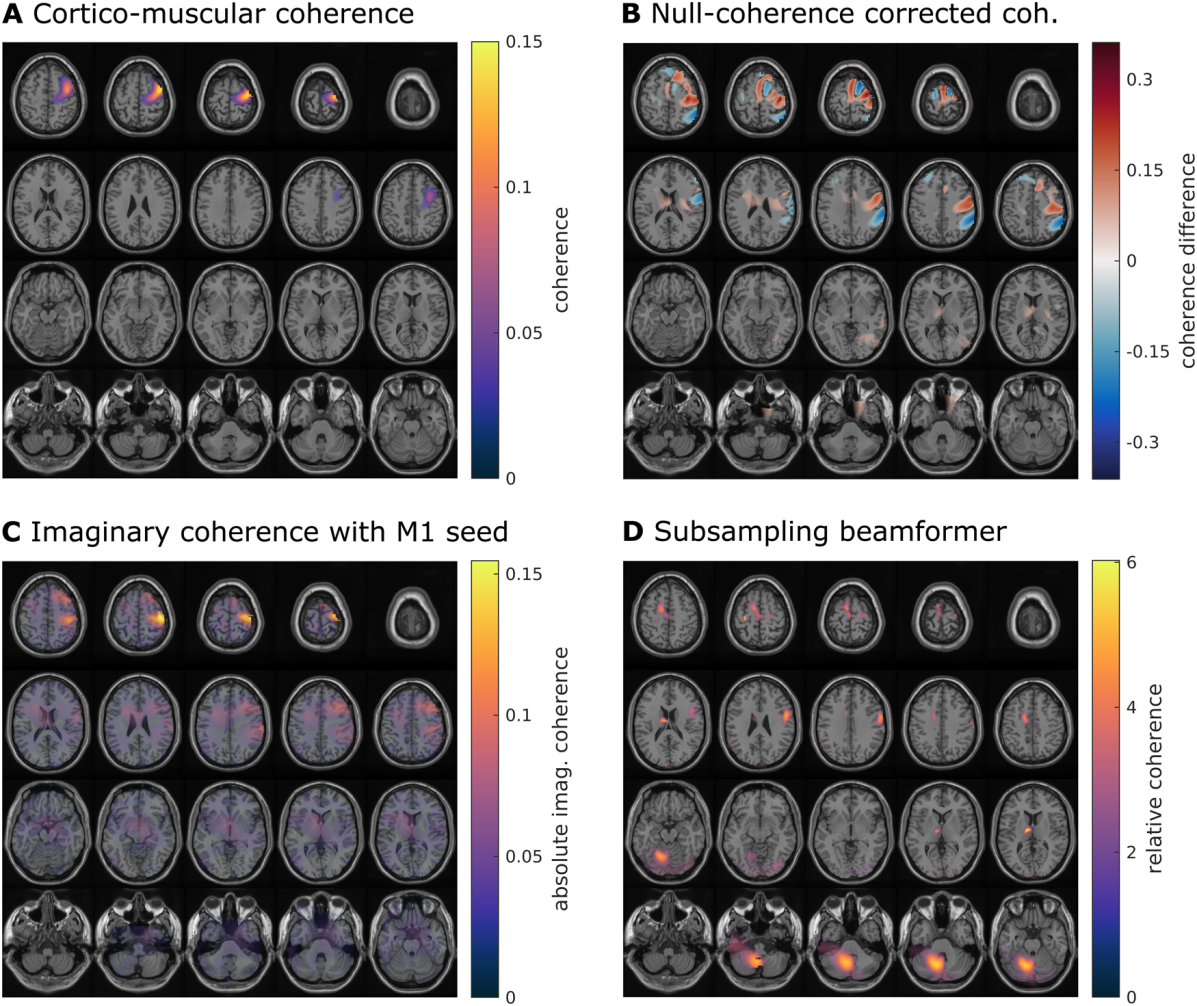
Real data analysis. (A) Cortico-muscular coherence as reported inSchoffelen et al. (2008). (B)Coherence that was corrected by subtracting the null coherence estimate.(C) Imaginary coherence (beamformer with the geometric correctionscheme) with a seed in primary motor cortex. (D) Coherence derived withthe subsampling beamformer, corrected by dividing through the nullcoherence estimates averaged across subsamples.

We used a cortico-muscular coherence dataset to test the subsampling approach onreal data.Figure 8shows the results fromthis analysis. We first identified the dipole with maximum CMC (Fig. 8A) as a seed for the subsequentanalyses. Next, we computed seeded cortico-cortical coherence difference withthe null coherence estimate (Fig. 8B) andthe imaginary part of coherency (Fig. 8C).The coherence difference shows a lot of spatial structure in the vicinity of theseed dipole, where the local maxima could reflect genuine maxima incontralateral sensorimotor, pre-motor, and supplementary motor areas. Yet, giventhe equal magnitude of the surrounding negative differences, and the similarspatial structure as observed in the reported side lobes (Schoffelen et al., 2008), these results may bespurious. Similarly, for the imaginary coherence, the focal maxima surroundingthe seed regions may reflect genuine interactions, but could just as well bespurious side lobes. In contrast to the two more traditional approaches, thesubsampling result clearly identify local maxima, distant to cM1, in theipsilateral cerebellum, ipsilateral M1, contralateral premotor cortex, possiblyin supplementary motor areas, and in the thalamus.

## 4 Discussion and Future Directions

Brain connectivity plays a central role in many prevalent hypotheses on brainfunctioning and organization (Bonnefond et al.,2017;Fries, 2005,^2015^;Jensen &Mazaheri, 2010). Thus, the estimation of functional connectivity based onelectrophysiological processes is a necessary tool for the experimental assessmentof those theories. Over the years, many different measures of brain connectivityhave been put forward (Aviyente et al., 2011;Ghanbari & Moradi, 2020;Vinck et al., 2011) and the methods to applythese have been refined (Dalal et al., 2006;Hillebrand et al., 2012;Kuznetsova et al., 2021;Nunes et al., 2020;Woolrich et al.,2011). Despite these efforts, results from non-invasive recordings,especially phase synchrony measures, have stayed sparse and methodologicalchallenges remain (Bastos & Schoffelen,2016;Colclough et al., 2016;He et al., 2019;Palva et al., 2018;Schoffelen & Gross, 2009). In this paper, we aimed at addressingsome of these challenges through a new beamformer-based connectivity estimationframework, which utilizes three key components: a two-dipole beamformer approach toestimate all-to-all connectivity, an estimation of the null coherence of the modelunder the assumption of no interaction, and a sensor array subsampling approach tofurther mitigate the influence of spatial noise. Our all-to-all approach ismotivated by the fact that—even in an unrealistically well-controlledcontrast—misspecification of the seed dipoles leads to spatial structure inconnectivity difference maps that can be mistaken for true interactions. Moreover,because experimental contrasts almost invariably contain differences in sourceactivations and SNR, difference maps of connectivity may show spatial structure thatis not due to changes in actual interaction between sources. For this reason, it isdesirable to estimate the spatial leakage of connectivity directly from the data. Weexplored the possibility to use such null coherence estimates, based on the weightedinner product between pairs of spatial filters. A two-dipole beamformer model ismotivated by the notion that beamformer estimates are distorted in the presence ofunderlying correlations. Furthermore, we propose to use sensor array subsampling inorder to smooth out the spatial noise at the benefit of the true interactions.

Some of the key components of our approach have been proposed before, in one form oranother, but mostly with a different intention, and were never combined for theassessment of connectivity. We compared the performance of our approach to otherall-to-all reconstruction schemes, which used only a subset—or none—ofthe key components.

Using an extensive set of simulations, we showed that our approach outperforms theother, often more traditional, all-to-all approaches tested. The overall detectionrate, specifically at physiologically meaningful interaction strengths and at a widerange of phase angles, was highest for the proposed subsampling based method. Thishigh detection rate was accompanied by the overall lowest false positive rate. Whileperformance was considerably affected when the relative source amplitude ofcompeting, non-interacting sources was increased (Supplementary Fig. S3), ourapproach still showed the overall lowest false positive rate (Fig. S5A). Based on theseobservations, we argue that the proposed reconstruction approach can be a promisingpipeline to be evaluated on real MEG data for the robust detection of phasesynchronization in brain networks.

In the following section, we compare our proposed method to a few prominent alreadyexisting methods which correct for spatial leakage in functional connectivity. Oneof the first methods for leakage correction is the proposal byNolte et al. (2004)to use the imaginary part ofcoherency. By discarding the real part of the signal, this method corrects forlinear leakage—but also misses any true zero-lag connectivity. Another classof approaches uses signal orthogonalization as a means to correct for leakage:Brookes et al. (2012)andHipp et al. (2012)introduced this method for pair-wiseseed-based comparisons andColclough et al.(2015)extended the method to several regions-of-interest (ROIs). Lastly,Wens et al. (2015)use a geometriccorrection scheme for the suppression of spatial leakage which models the spatialleakage as a point spread function. Our proposed approach differs on severaldimensions from the described methods. First, our approach allows for an estimationof all-to-all connectivity, which the orthogonalization methods do not provide:since they rely on partial correlations, even the ROI-method ofColclough et al. (2015)limits the achievable granularityas functionally overlapping ROIs need to be avoided. These methods thus also callfor the explicit specification of a seed or seed region. Further, they estimatesignal-amplitude correlations while our relies on phase differences. Second, somemethods such as the orthogonalization methods and imaginary coherence willpotentially over-correct the signal for true zero-lag connectivity. Our proposedmethod does not suffer from this, since the leakage correction is achieved via thetwo-dipole spatial filter. Lastly, none of the discussed methods deploys measures toreduce the variance of their estimate, which we achieve via the subsampling ofsensors.

### 4.1 Real data analysis

We then show that our approach can uncover biologically plausible coherencebetween the contralateral primary motor cortex and other motor areas in thebrain. The cerebellum and ipsilateral motor cortex were also identified using aCMC-only approach (Schoffelen et al.,2008). The other nodes identified putatively reflect the same networkas identified inGross et al. (2002),which used a different motor task than the one reported here. The observedpattern of functional connectivity was not revealed using the other, traditionalapproaches. In extension to our simulations, we found that a division as opposedto subtraction of the null coherence estimate in the subsampling case providedclean results. This is made possible since the averaging across many subsamplesstabilizes the null coherence estimate. We thus show that our subsamplingapproach works well on real data; the validation of the best correction scheme(division or subtraction) is beyond the scope of this paper as this cannot bedecided on a single real data set. Similarly, a validation of all-to-allconnectivity using our approach with subsequent statistical evaluation acrossparticipants is left for future research.

### 4.2 Limitations and further exploration

#### 4.2.1 Number of interacting dipoles

In our simulations, we only look at cases of two interacting dipoles. Onemight argue that this is a shortcut with respect to our use of thetwo-dipole beamformer (we remind the reader that the two-dipole model is notonly applied to the interacting dipoles of interest, but also allinteracting noise sources). To demonstrate that this approach also fareswell in different scenarios, we repeated the analysis for three interactingdipoles and present the results inSupplementary Figure S10. While the overall hit rategoes down, our method still outperforms the other algorithms in a comparablepattern toFigure 7. However, theseresults do not necessarily expand to more complex or distributed sourceconfigurations, which could be an interesting avenue for futureevaluation.

#### 4.2.2 Spatial resolution

In our approach, we increase robustness through sensor subsampling. Apossible limitation of this could be a decreased spatial resolution throughthe reduction of number of sensors per subsampling realization. However, asin ensemble learning approaches (Breiman,1996), we then aggregate the results across many randomsubsampling realizations, effectively using all sensors. Since our resultsyield direct information about the influence of dipole distance between theinteracting dipoles, we can inspect the results as a function of dipoledistance. InSupplementary Figure S11, we show that for dipoles that arefurther apart than around 1.5 cm, our proposed approach outperforms theother methods we tested. This further confirms that the subsampling ofsensors does not lead to a reduction in resolution, but indeed acts as a wayto minimize variance as proposed in ensemble learning (Breiman, 1996). Ultimately, however, we do notspecifically test for spatial resolution in this paper, as our focus lies onthe robust identification of (further apart) interacting sources incomparison to established methods. A detailed investigation of spatialresolution is left to future work.

#### 4.2.3 Background noise and scaling parameter

Our simulations used 18 active background dipoles to simulate brain noise. Tofurther investigate the impact of noise on our method, we conducted thesimulations with a noise covariance matrix estimated from resting-stateactivity of a real data set. The results of this simulation are presented inSupplementary FigureS12. The FROC curves show that the hit rate decreased for allalgorithms as compared toFigure 7, butthe two-dipole subsampling beamformer approach still clearly outperforms theother methods. From a theoretical point of view, some steps taken in ourapproach may clearly be a violation of reality. For instance, the assumptionthat the influence of noise in the null coherence bias estimation step canbe modeled with a single scaling parameter (which is equivalent to assuminga diagonal sensor noise covariance) is a rather large simplification. Futurework is needed in order to investigate in detail the limitations of thissimplification. Our preliminary control analysis, presented inSupplementary FigureS12, shows that the use of more realistic noise covariances isfeasible but does not seem required.

#### 4.2.4 Realistic coherence values

In the current simulation, we tested a broad range of coherence values,starting from a value of 0.2. Physiologically meaningful neuronalinteractions may lead to coherence values that are lower, in the range of0.1–0.2 (see, e.g.,Vezoli et al.,2021). We show that our approach outperforms the other methodsalso at a simulated coherence of 0.2, although the hit rate dropped to about50%. Based on these data, our approach will be useful to detect a largeportion of realistic interactions at the upper boundary, as furtherunderlined by our analysis of real data. The performance of our approach forlower coherence values—as well as method tweaks to improveperformance for low coherence values—is open for futureinvestigation.

### 4.3 Future work

We foresee future work to explore in more detail certain aspects of the proposedanalysis scheme. For instance, regarding the subsampling, we have settled on afixed number of subsampled reconstructions, using a random number of sensors(between 50 and 150 out of 275 sensors for the simulations and between 40 and120 out of 151 sensors for the real data), and combined the reconstructions bymeans of averaging. Although those parameter choices were motivated by initialexplorations, strategies to estimate the optimal number of sensors for thesubsampling, and different combinatorial strategies (e.g., by also taking thevariance structure across subsample-based reconstructions into account) mayfurther improve the performance of the subsampling based approach.

Regarding statistical testing, the two-dipole subsampling beamformer can outputz-scores based on the variance among the subsamples (*cf.*Fig. 3), which can be used for thresholdingand the comparison to the null coherence estimate, but also for furtherstatistical evaluation across data sets (e.g., statistical testing between oracross participant groups). Within-data set testing could be possible by notonly bootstrapping sensor subsets but also trial subsets. Our approach couldthus be further modified to include statistical testing schemes based on thesubsampling.

Lastly, future work could furthermore investigate if a similar approach is alsofruitful with distributed source reconstruction models and compare thesubsampling approaches to established distributed methods.

To conclude, we show a new beamformer-based connectivity estimation framework,which addresses some well-documented challenges of functional connectivity inelectrophysiology. We hope that our approach can be a useful tool in the studyof connectivity within basic and clinical neuroscience.

## Supplementary Material

Supplementary Material

## Data Availability

Code and simulation data are available athttps://github.com/schoffelen/shared_subsampling.

## Footnotes

1 Note that, as any analysis model, the two-dipole beamformer comes withmathematical assumptions, specifically that the reconstructed activity alwayspertains to activity in two dipoles at the same time and therefore leads toslightly different estimates of activity for a given location, depending on theother considered location. However, the aim here is to optimally reconstruct theinteraction between sources, and not the activity in a single source. Thus, thespatial filters may capitalize on different temporal aspects of single sources,since different aspects of the source activity may be coherent with differentconnected sources.

2 Note that the matrixNineq. 8reflects all signal contributions to the observeddata that are not originating from the two dipole pairs of interest, that is,$N=N_{i}+N_{s}$,where$N_{i}$stands for the sensor-projected interfering sources and$N_{s}$is the sensor noise. Consideringeq. 8,the last term can thus be written as:$w_{1}^{⊤}\left(N_{i}N_{i}^{⊤}+N_{i}N_{s}^{⊤}+N_{s}N_{i}^{⊤}+N_{s}N_{s}^{⊤}\right)w_{2}$.This illustrates that the noise terms only vanish ineq. 9if there are not more interfering sources thansensors and if these interfering sources are not correlated with the sources ofinterest (although one might argue that correlated interfering sources are bydefinition not interfering anymore). A similar line of reasoning applies to theterms ineq. 8that are related to thecorrelation between the two sources of interest and the noise.
